# Association Between Preoperative GLP-1 Receptor Analog Use and Postoperative Complications and Mortality Following Lumbar Fusion Surgery

**DOI:** 10.1177/21925682251391693

**Published:** 2025-10-25

**Authors:** Ahad A. Kesaria, Farhad A. Marzook, James M. Glover, Pouya Alijanipour

**Affiliations:** 1 John Sealy School of Medicine, 74950 The University of Texas Medical Branch, Galveston, TX, USA; 2 Department of Orthopaedic Surgery and Rehabilitation, 12338 The University of Texas Medical Branch, Galveston, TX, USA

**Keywords:** lumbar arthrodesis, lumbar fusion surgery, GLP-1 analogs, postoperative complications, postoperative outcomes

## Abstract

**Study Design:**

Retrospective Cohort Study.

**Objectives:**

This study evaluates the association between preoperative GLP-1 RA (glucagon-like-peptide-1 receptor antagonist) use and postoperative outcomes in patients undergoing lumbar fusion surgery.

**Methods:**

TriNetX database identified patients undergoing lumbar fusion within 20 years using Current Procedural Terminology (CPT). Patients were categorized by GLP-1 RA use within 1 year preoperatively. 1:1 propensity score match (PSM) balanced demographics and comorbidities including race/ethnicity, age, gender, hypertension, diabetes, obesity, nicotine dependence, sleep apnea, ischemic heart diseases, chronic kidney disease, acute kidney failure, mood disorders, asthma, chronic obstructive pulmonary disease, heart failure, alcohol dependence, anemia, and vitamin D deficiency. Primary outcomes were 1-year complications postoperatively. Chi-square analysis, risk ratios (RRs), 95% confidence intervals (CI), and *P*-values were calculated; significance was *P* < 0.05.

**Results:**

4331 patients using preoperative GLP-1 RA were propensity score-matched with 179,268 controls without GLP-1 RA use, resulting in 4331 patients in each cohort after matching. At 1 year, GLP-1 RA users had significant reductions in DVT (1.4% vs 2.3%, RR = 0.64, 95% CI [0.464-0.883], *P* = 0.0061), PE (1.1% vs 1.6%, RR = 0.689, 95% CI [0.476-0.997], *P* = 0.0466), sepsis (4.0% vs 5.0%, RR = 0.811, 95% CI [0.66-0.995], *P* = 0.0447), all-cause mortality (2.1% vs 4.6%, RR = 0.46, 95% CI [0.36-0.589], *P* < 0.0001), pneumonia (2.4% vs 3.3%, RR = 0.716, 95% CI [0.548-0.936], *P* = 0.0139), and pseudoarthrosis (8.9% vs 13.8%, RR = 0.642, 95% CI [0.564-0.732], *P* < 0.0001) compared to non-users.

**Conclusions:**

Preoperative GLP-1 RA use is associated with a reduction in postoperative complications following lumbar fusion surgery. Further research is necessary to elucidate the underlying mechanisms and evaluate long-term outcomes.

## Introduction

Lumbar fusion surgery has become an increasingly common procedure over the past few decades. Originally introduced by Dr Fred H Albee as a treatment for tuberculosis related deformities, spinal fusion surgery has since evolved to treat a variety of conditions.^1-4^ With advents in instrumentation and technique, surgeons have been able to expand its use to treat numerous ailments, including degenerative disease, deformities, trauma, tumors, infections, etc.^5^ Despite the success of lumbar fusion, it is not without its postoperative complications. These can include pseudoarthrosis, mortality, deep vein thrombosis, and poor wound healing, among others.^6,7^ Moreover, there is a lack of understanding regarding the time frame in which these complications can occur.^8,9^ As demand for lumbar fusion surgery continues to rise, it is imperative to understand and optimize these postoperative outcomes to reduce complications and improve patient recovery.

Over the past few years, glucagon-like peptide-1 receptor agonists (GLP-1 RAs) have gained immense popularity as a highly effective tool for managing both type 2 diabetes and obesity. Working by increasing insulin secretion, it helps maintain glycemic control and promotes weight loss.^10^ Obesity, in particular with patients undergoing lumbar spine surgery, is associated with poorer postoperative outcomes, and patients with a higher body mass index (BMI) are encouraged to undergo weight loss prior to surgery.^11,12^ GLP-RA’s have been FDA approved as a weight loss technique and studies have demonstrated approximately 5%-15% body weight reduction in patients using GLP-RAs.^13,14^ The growing burden of obesity and its relevant risks highlight the importance of incorporating the use of GLP-RAs to promote weight loss and enhance metabolic health.

GLP-1 RAs’ promising characteristics in improving perioperative care, with potential to reduce risks and improve surgical outcomes, make it an evolving adjunct to orthopedic procedures. With obesity rates on the rise, surgeons expect an increase in number of patients eligible for orthopedic procedures and possible improved outcomes with the implementation of GLP-1 RAs.^15^ However, few studies have explored the benefits of using GLP-1 RAs preoperatively in spinal surgery. This study evaluates the association between preoperative GLP-1 RA use and postoperative outcomes following lumbar fusion surgery, including medical complications (deep vein thrombosis, pulmonary embolism, sepsis, all-cause mortality, postprocedural hematoma, pneumonia, stroke, myocardial infarction, and acute kidney failure) and surgical complications (postoperative infection, osteomyelitis, cerebrospinal fluid leak, readmission, emergency department visit, pseudoarthrosis, and mechanical complications).

## Methods

TriNetX, a United States based healthcare research database, was queried to obtain deidentified patient data from electronic medical records across healthcare organizations (HCOs) in partnership with TriNetX. HCOs include institutions such as hospitals and private groups that participate in sharing patient records. Due to its deidentified nature, data obtained from TriNetX follows IRB regulations and policies, making it exempt from IRB review and individual patient consent. This study utilized data from the United States Collaborative network, which encompasses 68 HCOs, with data from 46 of the 68 HCO providers used in this study. Data was analyzed from December 31st, 2004 to December 31st, 2024.

Patient data was stratified and analyzed into two cohorts. Each cohort consisted of patients who underwent lumbar fusion surgery within the past 20 years, identified using Current Procedural Terminology (CPT) codes: 22612, 22614, 22630, 22632, 22633, and 22634. Cohort A included patients who had undergone lumbar fusion surgery and had documented use of glucagon-like peptide-1 receptor analogs (GLP-1 RAs) (National Library of Medicine: Anatomical Therapeutic Chemical Classification System: A10BJ/NLM:ATC:A10BJ) within 1 year prior to surgery. Cohort B included patients who underwent lumbar fusion surgery without prior use of GLP-1 RAs. The cohorts were then propensity matched based on demographic and medical factors.

Following 1:1 propensity matching, a chi-square test was conducted using the TriNetX software to calculate relative risk (RR) with confidence intervals (CI) and standard mean difference to compare outcomes between the two cohorts. Patient demographics were matched based on age at index, gender, race, and ethnicity. In the Medical Factors Diagnosis category, patients were matched based on essential (primary) hypertension, nicotine dependence, diabetes mellitus, sleep apnea, obesity, ischemic heart diseases, chronic kidney disease, acute kidney failure, mood disorders, asthma, other chronic obstructive pulmonary disease, heart failure, alcohol dependence, vitamin D deficiency, anemia, BMI, and HgBA1c characteristic(s) (Table 1).

**Table 1.** Demographics of Patients With Preoperative GLP-1 RA Use (Cohort A) vs Patients Without Preoperative GLP-1 RA Use (Cohort B) Before and After 1:1 Propensity Matching

		Before PSM				
Cohort	Demographic	Mean ± SD	Patients, n	% of cohort	*P*	SMD
A	Age at index	62.3 ± 10.1	4331	100	<0.001	0.178
B		60.0 ± 15.0	174 387	100		
A	Male		1859	42.9	<0.001	0.116
B			84 938	48.7		
A	Female		2300	53.1	<0.001	0.118
B			82 345	47.2		
A	White		2940	67.9	<0.001	0.130
B			128 682	73.8		
A	African American		562	13	<0.001	0.127
B			15 710	9		
A	Asian		79	1.8	0.965	0.001
B			3165	1.8		
A	American Indian		20	0.5	0.044	0.028
B			511	0.3		
A	Pacific Islander		18	0.4	0.069	0.025
B			470	0.3		
A	Other race		151	3.5	0.003	0043
B			4771	2.7		
A	Unknown race		561	13.0	0.084	0.026
B			21 078	12.1		
A	Not Hispanic or Latino		3155	72.8	0.021	0.036
B			124 225	71.2		
A	Hispanic or Latino		308	7.1	<0.001	0.070
B			9458	5.4		
A	Unknown ethnicity		868	20.0	<0.001	0.080
B			40 704	23.3		
A	Essential (primary) hypertension		3473	80.2	<0.001	0.676
B			86 553	49.6		
A	Nicotine dependence		799	18.4	0.003	0.045
B			29 186	16.7		
A	Diabetes mellitus		3451	79.7	<0.001	1.493
B			34 630	19.9		
A	Sleep apnea		1843	42.6	<0.001	0.652
B			25 396	14.6		
A	Obesity		2431	56.1	<0.001	0.868
B			30 859	17.7		
A	Ischemic heart diseases		1308	30.2	<0.001	0.362
B			26 627	15.3		
A	Chronic kidney disease (CKD)		968	22.4	<0.001	0.400
B			14 339	8.2		
A	Acute kidney failure		678	15.7	<0.001	0.274
B			12 269	7		
A	Mood disorders		1865	43.1	<0.001	0.424
B			40 984	23.5		
A	Asthma		1003	23.2	<0.001	0.322
B			19 467	11.2		
A	Other chronic obstructive pulmonary disease		560	12.9	<0.001	0.147
B			14 677	8.4		
A	Heart failure		599	13.8	<0.001	0.259
B			10 711	6.1		
A	Alcohol dependence		77	1.8	0.001	0.055
B			4512	2.6		
A	Vitamin D deficiency		1376	31.8	<0.001	0.519
B			19 446	11.2		
A	Anemia		1090	25.2	<0.001	0.305
B			23 145	13.3		
A	BMI	34.3 ± 6.5	3360	77.6	<0.001	0.677
B		29.9 ± 6.5	129 743	74.4		
A	HgBA1C	6.9 ± 1.5	3360	77.6	<0.001	0.546
B		6.1 ± 1.3	62 256	35.7		

		After PSM				
Cohort	Demographic	Mean ± SD	Patients, n	% of cohort	*P*	SMD
A	Age at index	62.3 ± 10.1	4331	100	0.071	0.039
B		62.7 ± 11.4	4331	100		
A	Male		1859	42.9	0.633	0.010
B			1881	43.4		
A	Female		2300	53.1	0.880	0.022
B			2293	52.9		
A	White		2940	67.9	0.212	0.027
B			2994	69.1		
A	African American		562	13	0.300	0.022
B			530	12.2		
A	Asian		79	1.8	0.873	0.003
B			81	1.9		
A	American Indian		20	0.5	0.303	0.022
B			14	0.3		
A	Pacific Islander		18	0.4	0.869	0.004
B			19	0.4		
A	Other race		151	3.5	0.401	0.018
B			137	3.2		
A	Unknown race		561	13	0.873	0.003
B			556	12.8		
A	Not Hispanic or Latino		3155	72.8	0.344	0.020
B			3194	73.7		
A	Hispanic or Latino		308	7.1	0.063	0.040
B			265	6.1		
A	Unknown ethnicity		868	20.0	0.915	0.002
B			872	20.1		
A	Essential (primary) hypertension		3473	80.2	0.285	0.023
B			3433	79.3		
A	Nicotine dependence		799	18.4	0.153	0.031
B			748	17.3		
A	Diabetes mellitus		3451	79.7	0.390	0.018
B			3483	80.4		
A	Sleep apnea		1843	42.6	0.572	0.012
B			1817	42.0		
A	Obesity		2431	56.1	0.914	0.002
B			2426	56.0		
A	Ischemic heart diseases		1308	30.2	0.038	0.045
B			1220	28.2		
A	Chronic kidney disease		968	22.4	0.080	0.038
B			901	20.8		
A	Acute kidney failure		678	15.7	0.015	0.052
B			598	13.8		
A	Mood disorders		1865	43.1	0.421	0.017
B			1828	42.2		
A	Asthma		1003	23.2	0.199	0.028
B			953	22.0		
A	Other chronic obstructive pulmonary disease		560	12.9	0.519	0.014
B			540	12.5		
A	Heart failure		599	13.8	0.016	0.052
B			524	12.1		
A	Alcohol dependence		77	1.8	0.451	0.016
B			68	1.6		
A	Vitamin D deficiency		1376	31.8	0.030	0.047
B			1283	29.6		
A	Anemia		1090	25.2	0.203	0.027
B			1039	24.0		
A	BMI	34.3 ± 6.5	3360	77.6	<0.001	0.144
B		33.3 ± 6.7	3386	78.2		
A	HgBA1C	6.9 ± 1.5	3360	77.6	<0.001	0.203
B		6.6 ± 1.4	3386	78.2		

PSM = propensity score matching; SMD = standardized mean difference.

Primary outcomes were evaluated within 1-year post-operation and were categorized as either medical or surgical complications. Medical complications included deep vein thrombosis, pulmonary embolism, sepsis, all-cause mortality, postprocedural hematoma, pneumonia, stroke, myocardial infarction, and acute kidney failure. Surgical complications included postoperative infection, osteomyelitis, cerebrospinal fluid leak, readmission, emergency department visit, pseudoarthrosis, and mechanical complications (Table 2).

**Table 2.** Medical Codes for Included Diagnoses, Procedures, Events, and Medications

Variable	Code
DVT	ICD-10: I82.4
PE	ICD-10: I26
Infection	ICD-10: T81.4
Osteomyelitis of vertebra	ICD-10: M46.2
Intracranial and intraspinal abscess and granuloma	ICD-10: G06
Sepsis	ICD-10: R78.81, A41
Mortality	Deceased, ICD-10: R99
Postoperative hematoma	ICD-10: G97.6, G97.61
CSF leak	ICD-10: G96.0
Readmission	CPT:1013699
ED visit	CPT: 1013711, 99281, 99284, 99283, 99285, 99282, 1013723
Pneumonia	ICD-10: J18
Stroke	ICD-10: I63
MI	ICD-10: I21
Pseudoarthrosis	ICD-10: M96
Acute kidney failure	ICD-10: N17
Mechanical complications	ICD-10: T84.4, T85.6, T84, T84.1, T84.3
Wound dehiscence	ICD-10: T81.31

DVT = deep vein thrombosis, PE = pulmonary embolism, CSF = cerebrospinal fluid, ED = emergency department, MI = myocardial infarction.

## Results

A total of 183 599 patients met the inclusion criteria from the TriNetX query, consisting of 4331 patients who underwent lumbar fusion surgery with preoperative GLP-1 RA use and 179 268 patients without preoperative GLP-1 RA use. After 1:1 propensity matching, 4331 patients were assigned to each cohort.

Following the propensity matching, the demographic and medical factor differences between the cohorts were minimal, as confirmed with chi-square analysis. The standard mean difference (SMD) was used to evaluate baseline balance, with an SMD of less than 0.1 indicating that the variables were well-matched. Patients who underwent lumbar fusion with preoperative GLP-1 RA use (Cohort A) had a mean age at index of 62.3 years, compared to a mean age of 62.7 years in patients who underwent lumbar fusion without preoperative GLP-1 RA use (Cohort B). Both cohorts were predominantly female, with 53.1% of Cohort A and 52.9% of Cohort B identifying with the female gender. After matching, the White race was the most prevalent in both cohorts, representing 67.9% or Cohort A and 69.1% of Cohort B (Table 1).

### Postoperative Outcomes

**Figure 1.**
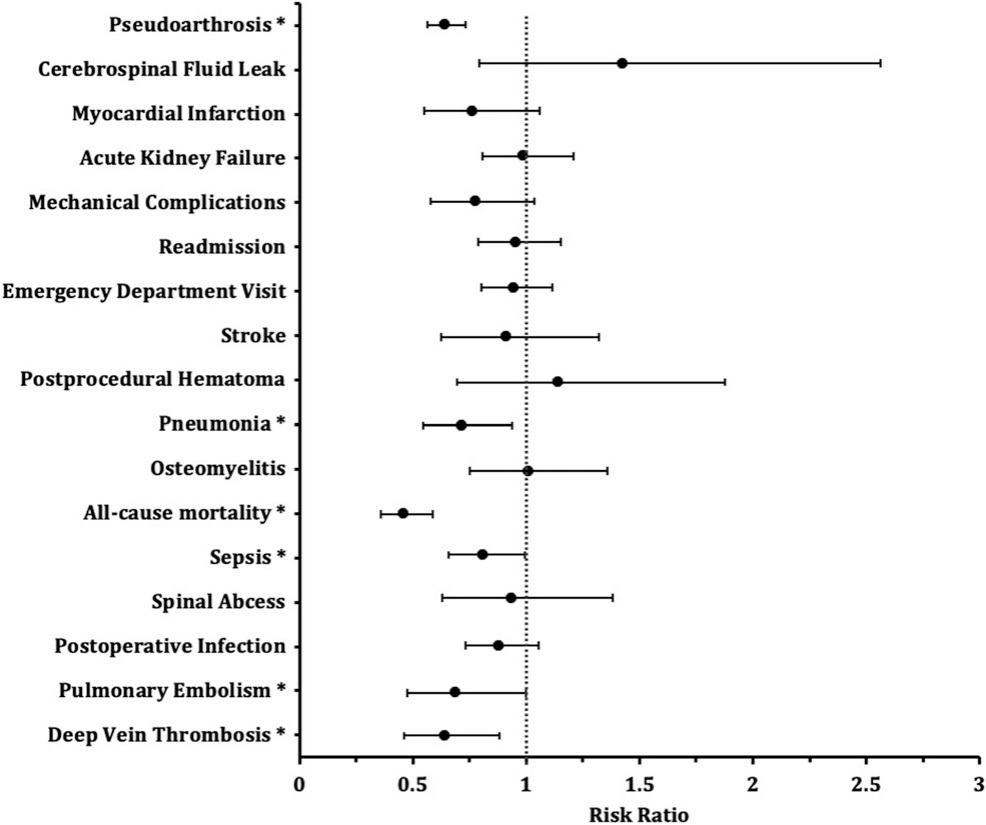
Risk Ratios of Postoperative Outcomes by GLP-1 Receptor Analog Use

Within 1 year postoperative, patients with preoperative GLP-1 RA use who underwent lumbar fusion surgery demonstrated significant reductions in DVT (1.4% vs 2.3%, RR = 0.64, 95% CI [0.464-0.883], *P* = 0.0061), PE (1.1% vs 1.6%, RR = 0.689, 95% CI [0.476-0.997], *P* = 0.0466), sepsis (4.0% vs 5.0%, RR = 0.811, 95% CI [0.66-0.995], *P* = 0.0447), all-cause mortality (2.1% vs 4.6%, RR = 0.46, 95% CI [0.36-0.589], *P* < 0.0001), pneumonia (2.4% vs 3.3%, RR = 0.716, 95% CI [0.548-0.936], *P* = 0.0139), and pseudoarthrosis (8.9% vs 13.8%, RR = 0.642, 95% CI [0.564-0.732], *P* < 0.0001) compared to non-users of GLP-1 RAs prior to surgery (Figure 1, Table 3).

**Table 3.** Outcomes Following Lumbar Fusion After 1:1 Propensity Matching of Patients With Preoperative GLP-1 RA Use (GLP-1 RA Users) vs Patients Without Preoperative GLP-1 RA Use (Control)

Outcome	GLP-1 RA users (N = 4331)	Control (N = 4331)	Risk ratio (95% CI)	*P-*value
1-year outcomes				
DVT	60	93	0.640 (0.464-0.883)	0.006*
PE	47	68	0.689 (0.476-0.997)	0.047*
Postoperative infection	205	233	0.878 (0.732-1.054)	0.163
Osteomyelitis	87	85	1.010 (0.751-1.357)	0.948
Intracranial and intraspinal abscess and granuloma	48	51	0.934 (0.631-1.382)	0.733
Sepsis	158	194	0.811 (0.660-0.995)	0.045*
All-cause mortality	90	196	0.460 (0.360-0.589)	<0.0001**
Postprocedural hematoma	33	29	1.141 (0.694-1.875)	0.603
CSF leak	27	19	1.427 (0.795-2.562)	0.232
Readmission	195	205	0.953 (0.788-1.154)	0.625
ED visit	243	257	0.946 (0.801-1.116)	0.508
Pneumonia	90	125	0.716 (0.548-0.936)	0.014*
Stroke	52	57	0.911 (0.627-1.323)	0.624
MI	63	83	0.765 (0.553-1.059)	0.105
Pseudoarthrosis	325	525	0.642 (0.564-0.732)	<0.0001**
Acute kidney failure	176	184	0.988 (0.808-1.209)	0.908
Mechanical complications	79	102	0.776 (0.580-1.038)	0.086

Categorical data presented as n (% of cohort).

**P < 0.0001; *P < 0.05.

DVT = deep vein thrombosis, PE = pulmonary embolism, CSF = cerebrospinal fluid, ED = emergency department, MI = myocardial infarction.

## Discussion

In this propensity score-matched cohort study, preoperative use of GLP-1 RA use was associated with significantly improved postoperative outcomes following lumbar fusion surgery. Patients experienced lower risks of venous thromboembolism (DVT and PE), major infections (sepsis and pneumonia), all-cause mortality, and pseudoarthrosis (fusion nonunion) within 1 year after surgery. In summary, patients on GLP-1 RAs before surgery had fewer life-threatening complications and a higher likelihood of solid arthrodesis.

Potential mechanisms behind the action of GLP-1 RA can explain why they played a major role in enhancing postoperative outcomes in lumbar fusion surgery. By promoting weight loss, GLP-1 RAs may decrease the strain a higher BMI places on cardiopulmonary systems. A study done by Willenberg et al^16^ demonstrates how increased abdominal pressure from obesity can induce a higher level of venous stasis in the lower limbs. This impaired venous function can explain why patients with a higher BMI undergoing lumbar fusion experience greater venous thromboembolism.^12,17,18^ Moreover, weight loss generally enhances mobility and rehabilitation after surgery which has been shown to decrease risk of complications and morbidities.^19^ These mechanisms of weight loss can help explain the association our study found in GLP-1 RAs reducing the risk of DVT and PE. In orthopedics, there remains an unchallenged assumption that a higher BMI increases the risk of DVT and PE following surgery.^20^ The results of this study suggest that the use of GLP-1 RAs could offer reassurance to surgeons, addressing these concerns and potentially shaping future practice to prevent these complications.

By promoting tighter glycemic control preoperatively, GLP-1 RAs reduce the risk of infections such as sepsis and pneumonia. Hyperglycemia is known to impair innate immunity, weakening the body’s ability to fight infections and heal wounds effectively.^21^ A study by Tang et al^22^ reported higher rates of postoperative pneumonia in hyperglycemic hip fracture patients. Similarly, another study found that perioperative hyperglycemia increased the likelihood of infection, such as sepsis, by nearly 7 fold in orthopedic trauma patients.^23^ Improving glycemic control in patients undergoing lumbar fusion can potentially help prevent the development of serious infections, such as pneumonia and sepsis. Future research is needed to further confirm the association between GLP-1 RAs and a reduced risk of these complications, through its mechanism of tightened glycemic control.

Pseudoarthrosis is one of the major complications following lumbar fusion and serves to represent the long-term success of the surgery. The use of GLP-1 RAs preoperatively was associated with a reduced risk of pseudoarthrosis, or fusion nonunion. Emerging evidence demonstrates that GLP-1 RAs can have a beneficial effect on bone formation, by promoting osteoblastic activity and inhibiting osteoclastic bone resorption.^24,25^ Moreover, weight loss induced by GLP-1 RA use can help offload stress on the spine, improving mobility and recovery.^19^ These mechanisms should be further examined to understand the near 30% reduction in nonunion risk seen in our GLP-1 RA cohort.

With the rise in lumbar fusion procedures being performed globally, there remains a persistent interest in understanding the risk of mortality associated with these surgeries. Although Salmenkivi et al^26^ found that mortality rarely occurs in spinal surgery, it is underscored that the risk is not zero and, therefore, steps should be taken to minimize complications and optimize patient outcomes. Additional studies further emphasize the paucity in literature on mortality trends in spinal surgery and the ongoing search for strategies to reduce complications.^27,28^ This gap in literature elicits the importance of interventions such as preoperative GLP-1 RA use in reducing mortality, as seen with our study, by almost half. Through further investigation behind the benefits of GLP-1 RAs, more long-term lumbar fusion outcomes can be evaluated and used to improve overall survival following surgery.

The impact of GLP-1 RA use on postoperative complications has been analyzed in other orthopedic procedures. Choudhury et al^29^ found that preoperative GLP-1 agonist use reduced the risk of mortality in patients without increasing other complications in patients who underwent total shoulder arthroplasty. Additionally, literature suggests that the preoperative use of GLP-1 RAs leads to a decrease in readmissions and prosthetic joint infections in patients who underwent total knee and total hip arthroplasty, respectively.^30,31^ Overall, the trend indicates that these medications are safe in a perioperative setting and potentially advantageous for reducing complications. The results seen in our study could be impactful in improving the quality of life and care for patients undergoing orthopedic and spinal procedures.

Preoperative optimization is crucial for improving surgical outcomes, particularly in managing excess body weight and hyperglycemia, which are significant risk factors for postoperative complications.^20,23^ Combination of traditional preoperative weight loss methods, such as diet and exercise, with GLP-1 RAs may offer a more comprehensive approach to improving surgical outcomes, however more studies are required on this subject. Recent multi-society guidance, including the American Society of Anesthesiologists, suggests that most patients may continue GLP-1 RA therapy prior to elective surgery, with individualized assessment of aspiration risk for those with symptoms or conditions that may delay gastric emptying. For higher-risk patients, the guidance notes that temporary discontinuation before surgery and resumption once gastrointestinal function has recovered may be appropriate. These considerations highlight the importance of balancing aspiration risk against the metabolic benefits of continued therapy in the perioperative setting.^32,33^ Future research should focus on identifying specific patient criteria for the clinical implementation of GLP-1 RA therapy into standard preoperative protocols. This approach could help reduce catastrophic complications, such as sepsis and mortality, while also improving the fundamental goal of surgery - achieving proper fusion. Further studies are needed to prove causation and to develop more refined guidelines on when and how GLP-1 RAs should be used before lumbar fusion.

While we believe this study provides valuable insights, it does possess certain limitations. Analyses using large databases such as TriNetX are limited to the accuracy of administrative coding data from participating HCOs, which can lead to potential misclassifications and residual confounding despite propensity-score matching. For example, both BMI and HgB A1C were not adequately matched between groups, representing a potential confounding bias in the interpretation of our results. Additionally, the retrospective nature of this study means that it is observational and cannot prove causation. Although several known risk factors for lumbar fusion complications were accounted for, it is possible that not all influential differences between the cohorts were fully controlled therefore residual confounding may remain. Important surgical factors such as operative year, hospital type, surgical approach, number of fused levels, operative time, and blood loss were not available and could not be incorporated, which may also substantially influence outcomes. Furthermore, compared to well-established databases like NIS and NSQIP, the composition and underlying structure of data within the TriNetX database are less well-documented, which may impact the interpretation and generalizability of findings. This study focused on 1-year outcomes, leaving longer-term effects unmeasured. There may have been a selection bias as patients on GLP-1 RAs may have had better access to healthcare, a higher socioeconomic status, and differing lifestyles, potentially influencing their medical surveillance and overall outcomes. Because we did not apply a correction for multiple comparisons, the possibility of a type 1 error exists. Finally, because outcomes were captured over a 1-year period, some later events may not have been directly related to the surgical procedure, such as pneumonia. Evaluating shorter-term outcomes in the future could help distinguish these surgery related events from unrelated medical occurrences. Future investigations should evaluate whether outcomes such as pseudoarthrosis and mortality differ when stratified by comorbidities including obesity and diabetes, as such subgroup analyses could provide valuable clinical insight. Despite these limitations, this study demonstrates the association between preoperative GLP-RA use and improved outcomes of lumbar fusion surgery.

## Conclusion

Preoperative use of GLP-1 RAs was associated with fewer postoperative complications following lumbar fusion surgery. Further research is necessary to elucidate the mechanisms behind these findings and evaluate long-term outcomes.

## Data Availability

Data is available upon request.*
